# New Aspects of Magnesium Function: A Key Regulator in Nucleosome Self-Assembly, Chromatin Folding and Phase Separation

**DOI:** 10.3390/ijms20174232

**Published:** 2019-08-29

**Authors:** Takashi Ohyama

**Affiliations:** 1 Department of Biology, Faculty of Education and Integrated Arts and Sciences, Waseda University, 2-2 Wakamatsu-cho, Shinjuku-ku, Tokyo 162-8480, Japan; ohyama@waseda.jp; Tel.: +81-3-5369-7310; Fax: +81-3-3355-0316; 2 Major in Integrative Bioscience and Biomedical Engineering, Graduate School of Science and Engineering, Waseda University, 2-2 Wakamatsu-cho, Shinjuku-ku, Tokyo 162-8480, Japan

**Keywords:** Mg^2+^, DNA self-assembly, nucleosome self-assembly, chromatin, phase separation, ES cell

## Abstract

Metal cations are associated with many biological processes. The effects of these cations on nucleic acids and chromatin were extensively studied in the early stages of nucleic acid and chromatin research. The results revealed that some monovalent and divalent metal cations, including Mg^2+^, profoundly affect the conformations and stabilities of nucleic acids, the folding of chromatin fibers, and the extent of chromosome condensation. Apart from these effects, there have only been a few reports on the functions of these cations. In 2007 and 2013, however, Mg^2+^-implicated novel phenomena were found: Mg^2+^ facilitates or enables both self-assembly of identical double-stranded (ds) DNA molecules and self-assembly of identical nucleosomes in vitro. These phenomena may be deeply implicated in the heterochromatin domain formation and chromatin-based phase separation. Furthermore, a recent study showed that elevation of the intranuclear Mg^2+^ concentration causes unusual differentiation of mouse ES (embryonic stem) cells. All of these phenomena seem to be closely related to one another. Mg^2+^ seems to be a key regulator of chromatin dynamics and chromatin-based biological processes.

## 1. Introduction

Magnesium is an essential metal element, and its ion is the second most abundant cation in human cells [1,2,3,4]. The average human stores ~25 g of the element, with ~65% in bones and ~32% in complexes with nucleic acids and proteins [5]. Magnesium plays various important roles in the structures and functions of nucleic acids, chromatin, and enzymes (Mg^2+^ is the most frequently used metal ion cofactor), as well as in the cell cycle, apoptosis, early embryonic development, and cell differentiation [1,2,3,4,5,6,7,8]. Thus, the uptake and efflux of Mg^2+^ are highly regulated to maintain appropriate levels for functions within a given cell or compartment [2].

The effects of metal cations and polyamines on nucleic acids or chromatin were extensively studied in the 1970s ([9] and references therein) and were mostly understood in terms of charge neutralization and ionic strengths. During the same period of time, tRNA was found to have tightly bound Mg^2+^ hydrate ions in its L-shaped structure [10,11], and Mg^2+^ was indispensable for the formation of the L-shaped structure. X-ray crystallographic studies and in vitro studies suggested that tertiary structure of tRNA cannot be stably maintained in the absence of Mg^2+^ [12,13,14] or Mg^2+^-interacting nucleotides [15,16]. Ribozyme was also found to require Mg^2+^ binding for the stabilization of its tertiary structure and catalytic activity [17,18]. Regarding DNA, some non-B structures also require Mg^2+^ for their conformational transitions and stability [19,20]. These three examples show that Mg^2+^ is an important component for molecular integrity. However, in these cases, the effect of Mg^2+^ is generated by its binding or coordination to specific sites. Thus, the effect is different from simple charge neutralization.

In 2007, a Mg^2+^-implicated novel phenomenon was reported. Physiological concentrations of Mg^2+^ allow dsDNA molecules to sense homology and induce those with identical sequences to selectively assemble with one another, even in the presence of different dsDNA molecules (hereafter, except for cases in which confusion may arise, this review refers to dsDNA simply as DNA, as per convention). This phenomenon was named “DNA self-assembly” [21], and it is independent of the conformation and mechanical properties. Interestingly, in 2013, nucleosomes were also found to have homology sensing ability, and those with identical DNA sequences preferentially associate in the presence of mM levels of Mg^2+^ ions [22]. This phenomenon was named “nucleosome self-assembly”.

**Figure 1:**
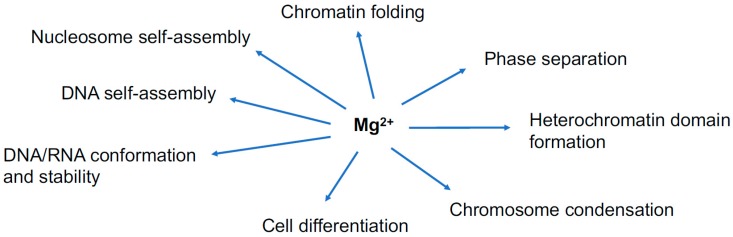
The current review sheds light on the implication of Mg^2+^ in the subjects or phenomena shown in the diagram.

The current review mainly describes the effects of Mg^2+^ on the higher-order structures of chromatin and chromosomes and the mechanisms underlying phase transitions, including the two self-assembling phenomena described above (Figure 1). All of these issues seem to be closely related to one another and involved in the dynamic regulation of chromatin infrastructure and function. The mechanisms underlying the formation of heterochromatin domains and overall chromatin folding seem to be particularly relevant. As another novel effect generated by Mg^2+^, this review touches on an unusual phenomenon occurring in the traits of mouse ES cells upon a subtle increase in the nuclear Mg^2+^ concentration [23].

## 2. Mg^2+^ Induces DNA Self-Assembly and Nucleosome Self-Assembly

The pairing of homologous chromosomes during the prophase of the first meiotic division is a well-known phenomenon. However, the mechanism by which each chromosome senses, recognizes, approaches, and interacts with its matching mate or homologue is a long-standing question. Recombination-dependent [24,25,26,27] and recombination-independent [28,29,30,31] mechanisms are known to participate in the homolog pairing. The difference between the two is whether the pairing requires programmed DNA double strand breaks (DSBs). In a similar phenomenon to the meiotic pairing, the pairing of homologous chromosomes also occurs in the non-meiotic cells of *Drosophila* and budding yeast [32,33,34]. The mechanisms of the recombination-independent homolog pairing and the “somatic pairing”, however, remain enigmatic.

If DNA molecules have the property of homology sensing and selective association among identical or nearly identical molecules, then this mechanism may explain the homolog pairing phenomena in meiosis and somatic cells. In relation to this issue, Kornyshev and Leikin proposed the following hypothesis: Two DNA fragments with homologous sequences can adopt an electrostatically favorable alignment by facing sugar-phosphate backbones (negatively charged) toward the major grooves (positively charged) over a large juxtaposition length. In contrast, the alignment of nonhomologous sequences will require higher energy for juxtaposition, and thus they cannot align [35]. Here, this hypothesis is referred to as the “strand-groove register hypothesis”, for convenience.

**Figure 2:**
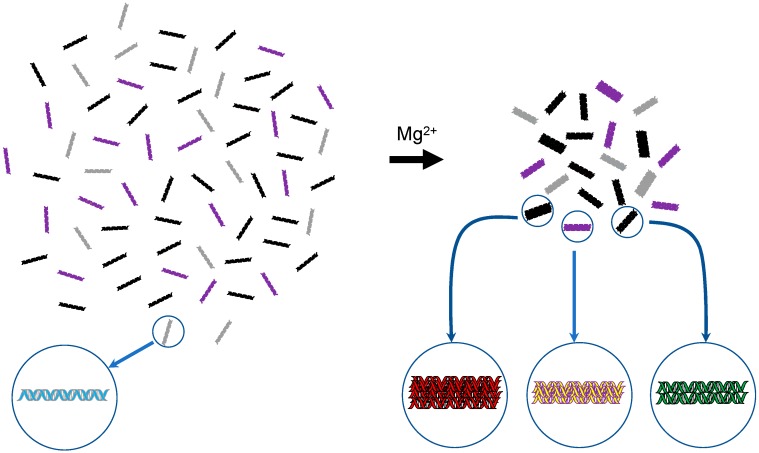
Schematic representation of DNA self-assembly. In 2007, it was found that dsDNA molecules with identical sequences preferentially interact with one another and assemble in the presence of physiological concentrations of Mg^2+^ [21]. Using different colors, the schema depicts an aqueous solution of four different DNAs on the left-hand side and their assemblages formed upon Mg^2+^ addition on the right-hand side. For the latter, the number of constituent molecules in an assemblage is unclear and thus a hypothetical image is drawn.

Several years after Kornyshev and Leikin proposed their hypothesis, experimental evidence was provided for the first time in 2007, showing that identical DNA molecules preferentially interact with one another and assemble. This phenomenon occurred when the DNA solution contained physiological concentrations of Mg^2+^ ions (Figure 2) [21]. This finding was obtained from electrophoretic analyses of the behaviors of DNA molecules, kinetic studies of DNA ligation reactions, and AFM (atomic force microscope) analyses. In the study, we used the term “DNA self-assembly” to describe the phenomenon, and thus in the current review, this term is also used. Importantly, it only refers to the assembly of identical dsDNA molecules. In 2008, DNA self-assembly was confirmed by another methodology. Baldwin et al. prepared two fluorescently tagged DNA molecules with identical nucleotide compositions and lengths, but different sequences, mixed them, and finally (after equilibration for two weeks) performed an image analysis by confocal microscopy. They observed the spontaneous segregation of the two kinds of DNA within each mixture (at the stage, the mixture had turned into a discrete liquid-crystalline aggregate (“spherulite”) [36]. In 2009, further confirmation of the phenomenon was obtained by using a parallel single molecule magnetic tweezers assay. Danilowicz et al. demonstrated that even in the absence of Mg^2+^ ions, homologous pairing of two DNA molecules occurs [37]. In 2014, an in vivo study using *Neurospora crassa* suggested the presence of the direct sequence recognition mechanism between identical DNA regions, in which 3 bp homology sequences with a matching periodicity of 11 or 12 bp seem to be involved in the direct recognition [38]. Thus, the intrinsic homology-based pairing interactions of DNA might be the “default option” in vivo [37]. Clarification of the underlying molecular mechanism will require further studies.

The important point that should first be taken into consideration is that eukaryotic genomes are packaged into chromatin. Accordingly, the homologous chromosome pairing occurs between supra-molecular architectures consisting of DNA, histones, non-histone proteins, and other associated molecules (i.e., not between “naked” DNAs). Although the linker DNA regions may be naked if DNA-binding proteins do not bind to these regions, the problem of steric hindrance must be overcome to allow even a single pair of two homologous linker DNA regions to associate. However, even though this hurdle could be cleared, it seems impossible that only a single pair of two homologous linker DNAs can connect two chromosomes. Instead, this process may be completed by employing many homologous pairs of linker DNAs. However, multiple juxtapositions or associations of DNA pairs located in different chromosomes seem to be topologically impossible. Thus, the explanation of the homologous pairing of chromosomes or chromatin fibers in terms of DNA self-assembly is apparently unreasonable.

To understand the mechanism underlying the pairing phenomena described above, the next question to be answered is whether nucleosomes, the building unit of chromatin, retain the DNA sequence-sensing property and can assemble between identical nucleosomes. In 2013, these questions were positively answered, in a phenomenon referred to as “nucleosome self-assembly”. This phenomenon, in which the identity of the nucleosomal DNA is sensed, is induced by Mg^2+^ ions [22]. Similar to the terminology used for the DNA self-assembly, nucleosome self-assembly only refers to the assembly occurring among nucleosomes with identical DNAs. In the current review, this terminology is also used. The details of nucleosome self-assembly will be described in another section. Although DNA occupies a considerable part of the nucleosome surface, the strand-groove register hypothesis obviously cannot be applied to nucleosomal DNAs with the coiled trajectory of the helical axis. Presumably, the nucleosome self-assembly occurs by some mechanism other than strand-groove registry.

## 3. Mg^2+^ Induces Chromatin Folding and Inter-Fiber Association

The extent of chromatin condensation is strongly influenced by the cationic conditions. According to Schwarz and Hansen, virtually all studies on the folding behavior of histone H1-depleted chromatin used solutions containing monovalent cations, before their study in 1994 [39]. Although monovalent cations can condense such chromatin at high concentrations, the condensation is limited, and the resulting fibers are not folded in an orderly manner (e.g., [40,41,42,43,44,45]). On the other hand, they showed that the divalent cation Mg^2+^ has a large effect on the condensation of H1-depleted chromatin fibers. Using histone octamers purified from chicken erythrocytes, they reconstituted nucleosomal arrays in vitro on the 12 tandem repeats of a 208 bp DNA fragment obtained from the *Lytechinus* 5S rDNA. The conformations of this array existed in an equilibrium between unfolded and highly folded states in the solutions containing Mg^2+^ ions at concentrations less than 2 mM. However, when the Mg^2+^ concentrations were greater than 2 mM, they observed a progressive shift of the equilibrium towards the formation of inter-fiber assemblies (irrespective of the folded or unfolded state of each fiber) [39,46]. In the interphase nucleus, Mg^2+^ and K^+^ seem to be required to form the porous (native) structure of the heterochromatin, which is apparently preserved in a condensed state in the presence of ≥ ~2 mM Mg^2+^ ions [47].

The effect of Mg^2+^ on chromosomes has also been extensively studied. The Mg^2+^ concentration has a strong effect on the condensed state of chromosomes [48,49,50,51]. Strick et al. performed an analysis with a three-dimensional high-resolution scanning ion microprobe and SIMS (secondary ion mass spectrometry) and reported that the condensed metaphase chromosomes require bound Mg^2+^ ions for their integrity [52]. Their analysis suggested that one Mg^2+^ was bound to every 20–30 nucleotides on diploid chromosomes. Then, what nanoscale-level changes are elicited with increasing or decreasing concentrations of Mg^2+^ in the chromatin fiber in a chromosome? Recently, this issue was examined by image analyses, using SEM (scanning electron microscopy) and STEM (scanning transmission electron microscope) tomography [51]. In the study, a reversible structural change between 11 nm and 30 nm chromatin structures in a chromosome was observed, according to the concentration of Mg^2+^ ions. When chromosomes were treated with buffer containing 5 mM Mg^2+^, they became more condensed, as compared to the treatment with buffer without Mg^2+^. The authors of this study suggested that Mg^2+^ ions may be a key determinant of the transformation between the 11 nm and 30 nm chromatin structures [51], although the presence of the 30 nm chromatin fiber itself is a matter of controversy [53].

The phenomena described above can be understood, in part, in terms of the charge neutralization: cations can neutralize the negative charge of DNA, which alleviates the electrostatic repulsion between DNA molecules, and this phenomenon seems to be valid even in chromatin and chromosomes. Thus, according to the increase in the Mg^2+^ concentration that can neutralize the DNA charge, the folding of chromatin fibers or inter-fiber associations should be facilitated. However, the reason why Mg^2+^ has such a great effect on chromatin condensation, as compared with monovalent and other divalent cations, is poorly understood. Obviously, the Mg^2+^ effect is not simply attributable to the difference in the ionic strength. The effect may be directly on chromatin [47]. Schwarz et al. explored the requirements for the inter-fiber association of oligonucleosomal arrays reconstituted in vitro and reported the following points: (i) H2A/H2B dimers are not implicated in the phenomenon, and (ii) when the nucleosomal arrays are trypsinized and their N- and C-terminal core histone tail domains are removed, Mg^2+^ cannot induce the association [46]. Based on these findings, they concluded that the inter-fiber association is directly mediated by the H3/H4 tail domains, through a non-Coulombic-based mechanism. However, considering that inter-fiber association was facilitated by the increase of Mg^2+^ concentrations (>2 mM) in their experiments, this cation is definitely indispensable for the phenomenon. The H3/H4 tail domains may be required only for “stabilization” of inter-fiber association.

The studies described above suggested that a Mg^2+^ concentration of ~2 mM may be the borderline. At concentrations less than ~2 mM, intra-fiber nucleosomal interactions are dominant. On the other hand, Mg^2+^ concentrations greater than ~2 mM induce inter-fiber association and fiber condensation. Then, how does a given single chromatin fiber become folded at Mg^2+^ concentrations less than ~2 mM? An in vitro study, performed to examine the assembling properties of nucleosomes in nucleosomal arrays, provided a partial answer to this question.

## 4. Self-Assembly among Identical Nucleosomes Occurs at Mg^2+^ Concentrations Less Than 2 mM

As described above, in the current review, the term nucleosome self-assembly means the assembly of the nucleosomes with identical DNA sequences. In 2013, a study using AFM-based analyses and a quantitative interaction assay revealed for the first time that nucleosomes with identical DNAs preferentially associate with one another in the presence of 0.2 to 1.5 mM Mg^2+^ ions [22]. Briefly, using a *Xenopus borealis* 5S rDNA nucleosome-positioning sequence [54], 601 and 603 sequences [55], and histone octamers purified from chicken erythrocytes, various homomeric or heteromeric octa- or tetranucleosomal arrays, or mononucleosomes were reconstituted in vitro. When heteromeric octa- or tetranucleosomal arrays were weakly induced to condense by 0.25 to 1.0 mM (for octanucleosomal arrays) or 0.2 to 0.5 mM (for tetranucleosomal arrays) MgCl_2_, the association between the same nucleosome species occurred predominantly, as compared to that between different species. Mononucleosomes also had the DNA-sensing and selective association properties at the 0.5 to 1.5 mM MgCl_2_ concentrations. In the experiments using octa- or tetranucleosomal arrays, inter-fiber association did not occur, in good agreement with the previous reports [39,46]. Many homologous pairing phenomena occur in cells, as described in the preceding section. The attractive force working between the identical DNA sequences and between identical nucleosomes may be used in the homologous pairing [21,22]. Furthermore, this homology sensing and assembling properties inherent in DNA and nucleosomes are presumably used in heterochromatin formation and may even trigger whole chromatin folding. This issue will be described in a later section.

## 5. Effects of Mg^2+^ on Linker DNA Conformation

The physical properties of DNA play a very important role in determining the nucleosome positions [56,57] and the paths of nucleosomal arrays [58]. In the latter, the flexibility of the linker DNA, which is a function of the nucleotide sequence [59], is deeply implicated. Importantly, DNA charge neutralization by cations increases its flexibility [60,61], resulting in the reduction of the spatial extensions of linker DNAs in chromatin because of the reduction of the persistence length [62,63]. Eventually, this change induces changes in the higher-order structure of chromatin, from an extended to folded state. This transition presumably generates various secondary effects on both the structure and function of chromatin.

Apart from the general effect of cations on DNA, Mg^2+^ also strongly influences the conformation and/or stability of some DNA structures [19,20,64,65,66]. Therefore, Mg^2+^ can give these effects on linker DNA conformation. Recently, an interesting phenomenon was reported: when A-tracts that form inward (AT-IN) bending were placed in a linker DNA, nucleosomal arrays containing such linkers consecutively formed highly compact structures [67,68]. This study clearly showed the importance of the linker DNA conformation in chromatin folding. This group also reported that extent of compaction of AT-IN arrays was further increased by the addition of 150 mM NaCl and 1 mM MgCl_2_. Some curved DNA structures are known to undergo conformational changes upon Mg^2+^ addition [64,65]. Therefore, the same effect may have been generated. Incidentally, the local chromatin conformation is also closely related to the gene activation mechanism [69,70].

The effect of Mg^2+^ on the linker DNA conformation is reminiscent of DNA methylation, which can also change the DNA conformation [71,72,73]. Mg^2+^ ions and DNA methylation may collaborate to form a specific local chromatin structure and prepare the infrastructure required for epigenetic gene regulation. However, at present, the effect of Mg^2+^ ions on the conformation or physical properties of methylated DNA is poorly understood.

## 6. Mg^2+^ and Phase Separation

There are many membrane-less compartments in cells. How these structures are autonomously constructed in a positionally and/or temporally regulated manner has been a long-standing enigma in biology. Recent studies suggested that phase separation, a well-known concept in polymer physics, is the driving principle underlying the formation of such structures [74,75,76]. The relationship between Mg^2+^ and phase separation is discussed in this section.

Cells have a class of membrane-less compartments that contain high concentrations of protein and RNA, known as ribonucleoprotein (RNP) granules/bodies [77,78,79,80]. Before moving on to the section theme, at first, the properties and functions of the RNP bodies will be briefly summarized. In 2009, Brangwynne et al. showed for the first time that the P granules of *Caenorhabditis elegans* have liquid-like properties, including fusion, dripping, and wetting [81]. The *C. elegans* P granules were originally found as cytoplasmic granules that are unique to the germ-line cells throughout the life cycle of the organism and were named in 1982 [82]. They seem to function in RNA metabolism or posttranscriptional regulation, to preserve the identity and special properties of germ cells [83,84]. In 2011, the nucleolus was also shown to have liquid-like properties [85]. The main function of the organelle is ribosome biosynthesis, and it is the largest RNA body.

Many other RNP granules/bodies are thought to have liquid-like properties as well. Examples include processing bodies (P bodies) (putative function: translational repression and/or RNA decay), stress granules (contribute to the regulation of gene expression), neuronal granules (contribute to the regulation of transport and local translation of dendritic mRNAs), Cajal bodies (function: modification of snRNA and small nucleolar RNA, etc.), and paraspeckles (function: sequestration of RNA or protein molecules) [86,87,88,89,90,91,92,93,94,95,96,97,98]. Although these granules/bodies have liquid-like properties, they form distinct environments in cells and facilitate chemical or biological reactions [74].

The RNP granules/bodies described above appear to be formed through liquid–liquid phase separation (LLPS) [74,75,76]. The important variables in LLPS are the concentrations of proteins and RNAs. Especially, the high local concentrations of modular interaction domains and/or intrinsically disordered, low complexity sequence (LCS) domains on RNPs are suggested to play a crucial role in the LLPS [99,100,101,102,103,104,105]. Furthermore, the salt concentration and temperature are also strong parameters in the LLPS, because they affect the free energy of the given system [101,106,107,108,109].

Heterochromatin domains, in which the major components are DNA, histones, and heterochromatin protein 1 (HP1), may be regarded as membrane-less compartments in the nucleus. Recently, Strom et al. proposed a model in which the LLPS drives the formation of the heterochromatin domain [110]. The background data were obtained from several in vitro and in vivo experiments; e.g., in the presence of low levels of Na^+^, highly concentrated aqueous solutions of the *Drosophila* HP1a protein showed spontaneous demixing and droplet formation at 22 °C in vitro, and these droplets reversibly dissolved at 37 °C and HP1a nucleated into liquid-like foci in vivo in the first stages of heterochromatin domain formation [110]. Using human HP1α protein, another group also reported the LLPS-based heterochromatin formation [111]. A low salt concentration appears to be an important parameter in LLPS and the stability of membrane-less compartments [102,107]. Accordingly, low Mg^2+^ levels are supposed to function similarly in vivo, in the formation of the heterochromatin domain. In conjunction with this, an attractive hypothesis was recently proposed by Wright et al. in which the phase separation dynamics occurring in the nucleus may be regulated by the ATP and free Mg^2+^ concentration balance or their levels [112].

ATP, which functions as “the major energy currency” in living cells, also functions as a chelator of Mg^2+^. In cells, most of the Mg^2+^ ions are chelated by ATP and other physiological chelators [113]. Thus, the hydrolysis of Mg^2+^-chelating ATP may increase the free Mg^2+^ concentration in cells. Indeed, the level of free Mg^2+^ in cells is reportedly increased via the hydrolysis of Mg^2+^-chelating ATP, at least at the metaphase stage, which correlates well with the timing of mitotic chromosome condensation, suggesting that the increase of free Mg^2+^ induces the condensation [114]. Now, let us return to the issue of heterochromatin domains in the nucleus. In the case of LLPS, increase of the free Mg^2+^ concentration is thought to be unfavorable [102,107,112]. On the other hand, chromatin condensation requires an increased Mg^2+^ concentration, as described in the preceding section. How are these mutually opposed requirements fulfilled in the heterochromatin compartments? The phenomenon of heterochromatin domain formation may not be understood by considering only LLPS and the “classic” knowledge on the relationship between chromatin condensation and Mg^2+^ or cation concentrations. The nucleosome self-assembly [22] may also be an important parameter in the heterochromatin domain formation.

## 7. Mg^2+^ and Repetitive DNA Folding and Phase Separation

Considerable portions of higher eukaryotic genomes are occupied by repetitive DNA sequences. For example, these sequences account for 45% and 52.5% of the mouse and human genomes, respectively [115]. Furthermore, highly repetitive DNA sequences (they comprise up to 10% in the case of the human genome) form constitutive heterochromatin that remains in the condensed form in most stages of the cell cycle, and its distribution is recognized as a definitive pattern in a given karyotype [116,117,118,119].

Repetitive DNA sequences may play an important role in the folding of genomes rich in these sequences. When we found the phenomenon of DNA self-assembly, we raised the possibility that this property of DNA may be used in the folding of repetitive DNA regions in genomes [21]. However, one problem was how DNA self-assembly overcomes the nucleosome structures. This problem was eventually solved by the discovery of nucleosome self-assembly six years later [22] (i.e., nucleosomes themselves can sense the identity of their DNA sequences and those with identical DNA associate with one another). Importantly, Mg^2+^ facilitates or enables DNA self-assembly and nucleosome self-assembly.

**Figure 3:**
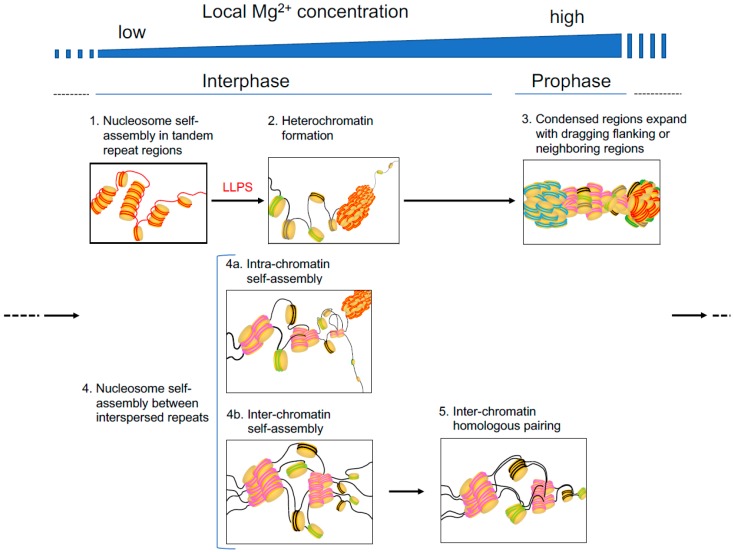
Hypothetical steps in Mg^2+^-induced and nucleosome self-assembly-implicated chromatin folding, condensation, or pairing in interphase and prophase. In the illustration, lines and thick disks indicate DNA and the histone core, respectively. Tandem repeats and interspersed repeats are indicated in red and dark blue (the former) and pink (the latter) lines, respectively.

Recently, Tang integrated these self-assembling phenomena and more recent studies including LLPS phenomena into a hypothesis: the interactions occurring among repetitive DNAs may drive the phase separation of these regions from the other chromatin regions [120]. The important point in this hypothesis is that the DNA is the main actor in the phase separation, which is in contrast to the LLPS mechanisms driven by protein interactions and/or protein-RNA interactions. In other words, repetitive DNA sequences may function as key chromosomal packaging modules [121,122]. Furthermore, a recent study using Hi-C (high-throughput chromosome conformation capture) data suggested the inter-chromosomal co-localization of several repetitive DNA sequences, especially those of the SINE (short interspersed element) family, in *Drosophila*, mouse, and human nuclei [123]. Repetitive DNA is divided into two types: tandem repeats and interspersed repeats. Here, the following hypothesis is raised. Tandem repeats may function in heterochromatin formation via Mg^2+^-induced nucleosome self-assembly and LLPS, while interspersed repeats may be used to connect distant regions harboring nucleosomes with identical DNA sequences or pair homologous interphase chromosomes via Mg^2+^-induced nucleosome self-assembly only (Figure 3).

## 8. Elevation of the Intranuclear Mg^2+^ Concentration Causes Unexpected and Unusual Differentiation of Mouse ES Cells

To determine the roles of Mg^2+^ in cell functions, the influence of changes in the Mg^2+^ levels has been widely studied, using various types of cells [2,4,7,124,125,126,127,128,129,130,131]. In these studies, the intracellular Mg^2+^ concentrations were changed by regulating its concentration in the growth media. However, to assess the direct causal relationship between the change in the “intranuclear” Mg^2+^ concentration and the resulting cellular traits, the regulation of the Mg^2+^ concentration in the media is indirect. Therefore, the microinjection of Mg^2+^ solutions into nuclei would be a powerful and direct method, if the control experiments are carefully performed to exclude the possible effects of the stimuli caused by the injection itself and the transient increase of the nuclear volume.

Recently, using various concentrations of mono-, di-, and polyvalent cation solutions and a microinjection technique, the effect of the elevated concentration of each cation in mouse ES cell nuclei was examined [23]. In the study, over 40 different solutions were prepared, and the microinjection was repeated more than 250 times for each solution. This experiment showed that only 2.7 mM, 18 μM, and 4.5 μM increases of the Mg^2+^, spermine, and spermidine concentrations, respectively, could differentiate a certain population of the cells into trophectoderm (the first cell type to appear during mammalian embryogenesis) or endoderm, even in media for the ES cell culture [23]. Incidentally, it is known that the trophectoderm is a lineage that mouse ES cells do not normally generate. These concentrations were those calculated by assuming that the injected solution had completely diffused within the nucleus, without leaking into the cytoplasm. Using the same assumption, the nuclear volume increase by the microinjection was only 2%. These values are just rough estimations. Although the underlying mechanism was not clarified, considering the finding that Mg^2+^ and polyamines acted similarly, some changes of the chromatin and DNA were speculated to cause the phenomenon. Regardless of the hypothesis, the analytical system using microinjection and ES cells may be advantageous for exploring the functions of low molecular weight substances, including Mg^2+^, in the processes occurring within the nucleus.

## 9. Conclusions

Mg^2+^ influences the higher-order structures of chromatin and chromosomes, and the mechanisms underlying phase separation, including the heterochromatin domain formation. Furthermore, DNA self-assembly and nucleosome self-assembly are facilitated or enabled by a certain range of Mg^2+^ concentrations. All of these phenomena seem to be closely related to one another and are presumably used in the dynamic regulation of chromatin infrastructure and genetic events in vivo. Thus, Mg^2+^ seems to be a key regulator of chromatin dynamics and chromatin-based biological processes. Substantiating this possibility is the next frontier in the research on chromatin dynamics and functions. Another issue is to elucidate how the local free Mg^2+^ concentration levels are regulated in the nucleus. These studies will clarify the cell traits that are considered to originate in chromatin or chromosomes, such as the underlying mechanism of the Mg^2+^-induced compulsory differentiation of mouse ES cells into trophectoderm. We have just entered an exciting new phase in Mg^2+^-chromatin research.
